# Clinicopathologic Predictors of Survival in Patients with Desmoplastic Melanoma

**DOI:** 10.1371/journal.pone.0119716

**Published:** 2015-03-26

**Authors:** Dale Han, Gang Han, Xiuhua Zhao, Nikhil G. Rao, Jane L. Messina, Suroosh S. Marzban, Amod A. Sarnaik, C. Wayne Cruse, Vernon K. Sondak, Jonathan S. Zager

**Affiliations:** 1 Department of Surgery, Yale University School of Medicine, New Haven, Connecticut, United States of America; 2 Department of Biostatistics, Yale University School of Public Health, New Haven, Connecticut, United States of America; 3 Department of Biostatistics, Moffitt Cancer Center, Tampa, Florida, United States of America; 4 Department of Radiation Oncology, Florida Hospital, Orlando, Florida, United States of America; 5 Department of Cutaneous Oncology, Moffitt Cancer Center, Tampa, Florida, United States of America; 6 Departments of Pathology and Cell Biology and Dermatology, University of South Florida Morsani College of Medicine, Tampa, Florida, United States of America; 7 Departments of Oncologic Sciences and Surgery, University of South Florida Morsani College of Medicine, Tampa, Florida, United States of America; University of Tennessee, UNITED STATES

## Abstract

**Background and Objectives:**

Desmoplastic melanoma is a unique subtype of melanoma which typically affects older patients who often have comorbidities that can adversely affect survival. We sought to identify melanoma-specific factors influencing survival in patients with desmoplastic melanoma.

**Methods:**

Retrospective review from 1993 to 2011 identified 316 patients with primary desmoplastic melanoma. Clinicopathologic characteristics were correlated with nodal status and outcome.

**Results:**

Fifty-five patients (17.4%) had nodal disease: 33 had a positive sentinel lymph node biopsy and 22 developed nodal recurrences (no sentinel lymph node biopsy or false-negative sentinel lymph node biopsy). Nodal disease occurred more often in younger patients and in cases with mixed compared with pure histology (26.7% vs. 14.6%); both of these variables significantly predicted nodal status on multivariable analysis (p<0.05). After a median follow-up of 5.3 years, recurrence developed in 87 patients (27.5%), and 111 deaths occurred. The cause of death was known in 79 cases, with 47 deaths (59.5%) being melanoma-related. On multivariable analysis, Breslow thickness, mitotic rate ≥1/mm^2^ and nodal status significantly predicted melanoma-specific survival (p<0.05).

**Conclusions:**

Nodal status predicts melanoma-specific survival in patients with desmoplastic melanoma. However, since patients with desmoplastic melanoma represent an older population, and a considerable proportion of deaths are not melanoma-related (40.5%), comorbidities should be carefully considered in making staging and treatment decisions in this population.

## Introduction

Desmoplastic melanoma (DM) is characterized by malignant spindled melanocytes within an abundant collagenous/myxoid (“desmoplastic”) stroma and is classically divided into histologic pure and mixed subtypes based on the extent of desmoplasia [1–3]. DM represents <4% of all primary cutaneous melanomas and was first reported by Conley et al., who described a melanoma variant with a relatively high potential for recurrence and aggressive clinical behavior [1, 4–6]. In that initial study of 7 DM patients, 3 developed nodal metastases (42.9%) and 4 (57.1%) died from DM [6].

Later studies indicated that DM most commonly develops on the head and neck of older males and often presents as a thicker tumor compared with conventional or non-desmoplastic melanoma [1–3, 5, 7–11]. In contrast to the initial findings of Conley et al., subsequent studies have not reported the high rates of nodal metastasis and melanoma-related death for DM patients [1, 2, 4–8, 10–13]. The true rates of nodal metastasis and melanoma-related mortality from DM, however, remain undefined. Recently reported nodal metastasis rates for DM range from 0 to 18.8%, with most studies describing lower nodal metastasis rates for DM compared with non-DM cases. In particular, pure DM cases exhibit a lower rate of nodal metastasis despite greater median tumor thickness [7–20]. Some studies suggest that survival for DM patients is similar to or better than what is observed for non-DM patients and that histologic subtype may influence survival [1–5, 8, 10, 12, 13].

The inconsistent reports on factors influencing nodal involvement and survival in DM patients make it difficult to assess prognosis and optimize staging and treatment of this disease. This is further complicated by the fact that DM typically affects older patients who often have comorbidities that adversely affect expected survival. We sought to address these issues by examining our single-institution experience with DM to identify melanoma-specific factors that influence nodal metastasis and survival in this population.

## Materials and Methods

After obtaining Institutional Review Board approval, a retrospective review was conducted from 1993 to 2010 to identify patients who were seen at Moffitt Cancer Center with a diagnosis of DM. This was a retrospective review with minimal risk to patients. The study was determined to be exempt from needing informed consent and was approved by the Institutional Review Board Committee at the University of South Florida. Patients were included in the study if they presented only with local disease at initial evaluation of a primary lesion at Moffitt Cancer Center or at an outside facility; patients who initially presented with clinical evidence of nodal or distant metastasis were excluded. Demographic, clinical, pathology and outcome data were reviewed.

Surgical treatment of primary tumors, with or without sentinel lymph node biopsy (SLNB), was performed either at Moffitt Cancer Center or at referring institutions. Primary tumors were resected with appropriate margins based on Breslow thickness. Patients with positive margins were treated with further resection unless further excision was not feasible. In these cases, patients were treated with radiation therapy (RT). Patients with a positive SLNB were routinely offered completion lymph node dissection (CLND). Node-positive patients included positive SLNB patients and patients who developed a nodal recurrence, who either had no SLNB or had a false-negative SLNB. Patients with thicker lesions (>4 mm) and lesions with neurotropism or positive margins where further resection was not feasible were evaluated for radiation therapy.

All patients seen at Moffitt Cancer Center underwent pathologic review of biopsy and surgical material from within and outside the institution by a faculty dermatopathologist. Primary tumor characteristics were routinely graded after 2004 and re-reviewed in cases with available tissue by a single dermatopathologist (JLM); in pre-2004 cases with available tissue, these characteristics were assessed upon re-review (JLM). In a total of 193 cases, pathology was able to be re-reviewed. The 2004 MSKCC classification system was used to characterize DM lesions as pure or mixed [2, 3]. Pure DM was defined as a spindle cell melanoma with ≥90% desmoplasia, while mixed DM was defined by desmoplasia involving <90% but >10% of the melanoma. Lesions were considered positive for perineural invasion (PNI) if tumor cells were found within the perineurium, or extensively surrounding and distorting cutaneous nerves. Mitotic rate (MR) was recorded per mm^2^, with cases grouped as ≥1/mm^2^ or <1/mm^2^ [21]. Sentinel nodes were evaluated by H&E and S-100 immunostaining routinely, and MART-1 immunostaining if indicated. CLND specimens were evaluated by H&E staining and immunostaining if indicated for suspicious or equivocal findings.

Statistical Analysis: Kaplan-Meier’s product limit approach was used to evaluate recurrence-free survival (RFS), overall survival (OS) and melanoma-specific survival (MSS). Evaluation began from the date that the primary lesion was diagnosed as DM to the date of first recurrence for RFS, to the date of death from any cause for OS and to the date of death from melanoma for MSS. Continuous variables were categorized using their medians or clinically relevant cutpoints. In particular, older age was defined by a cutpoint of ≥70 years of age. Log-log transformation was used with Kaplan-Meier curves to compute estimated 5-year survival probabilities and 95% confidence intervals (95% CI). Log-rank test was used to compare the levels of significant categorical variables for RFS, OS and MSS. Univariable and multivariable Cox’s proportional hazard regression models were implemented to model the effects of independent variables on the hazard rate. In comparing node-positive and node-negative patients, the Wilcoxon Rank-Sum test was used to compare age and Breslow thickness while Pearson’s chi-square test or Fisher’s exact test was used to test the independence between categorical variables. Univariable and multivariable logistic regression models were constructed to describe nodal positivity with estimated odds ratio (OR) of independent variables (e.g. age and histologic subtype). A p-value ≤0.05 was considered statistically significant. All statistical analyses were performed using Statistical Analysis System (SAS) software, version 9.3.

## Results

### Patients

Overall, 316 patients met inclusion criteria and were included in this study. Median follow-up was 5.3 years. Median age was 68.3 years (Table 1), the majority of patients were male (72.2%) and the majority of tumors were located on the head and neck (53.2%). Median Breslow thickness was 3.8 mm; in 7 cases (2%), thickness was unknown. Histologic subtype was evaluated in 193 cases (61.1%) with 90 of those (46.6%) classified as mixed and 103 (53.4%) classified as pure. In total, there were 55 node-positive patients (17.4%), comprised of 33 positive SLNB patients, 8 false-negative SLNB patients who developed a nodal recurrence in the draining regional nodal basin and 14 patients who never underwent a SLNB but developed a regional nodal recurrence. A CLND was performed in 28 of 33 positive SLNB cases, while 5 patients refused CLND or were lost to follow-up. Overall, 114 patients received RT to the primary resection site with 5 patients also having RT to regional nodal basins.

**Table 1:** Clinicopathologic characteristics correlated with nodal status in patients with clinically localized desmoplastic melanoma.

	All patients	Node Positive	Node Negative	p-value
**N**	**316**	**55**	**261**	
Age (years)				**0.01** ^**a**^
Median	68.3	62.6	69.4	
Range	15.8–95.8	34.6–86.2	15.8–95.8	
Gender				NS
Male	228 (72.2%)	42 (76.4%)	186 (71.3%)	
Female	88 (27.8%)	13 (23.6%)	75 (28.7%)	
Breslow thickness (mm)				**<0.01** ^**b**^
Median	3.80	4.65	3.60	
Range	0.5–35	1.2–18	0.5–35	
Clark level				NS
II	1 (0.3%)	0	1 (0.4%)	
III	12 (4.0%)	0	12 (4.8%)	
IV	146 (48.7%)	29 (55.8%)	117 (47.2%)	
V	141 (47.0%)	23 (44.2%)	118 (47.6%)	
Unknown	16	3	13	
Tumor location				NS
Head and neck	168 (53.2%)	26 (47.3%)	142 (54.4%)	
Extremity	75 (23.7%)	13 (23.6%)	62 (23.8%)	
Trunk	73 (23.1%)	16 (29.1%)	57 (21.8%)	
Histologic subtype				**0.03** ^**a**^
Mixed	90 (46.6%)	24 (61.5%)	66 (42.9%)	
Pure	103 (53.4%)	15 (38.5%)	88 (57.1%)	
Unknown	123	16	107	
Margin status				NS
Positive	40 (13.2%)	8 (15.7%)	32 (12.7%)	
Negative	262 (86.8%)	43 (84.3%)	219 (87.3%)	
Unknown	14	4	10	
Ulceration				NS
Yes	54 (21.9%)	13 (30.2%)	41 (20.1%)	
No	193 (78.1%)	30 (69.8%)	163 (79.9%)	
Unknown	69	12	57	
Mitotic rate				NS
≥1/mm^2^	97 (47.1%)	21 (58.3%)	76 (44.7%)	
<1/mm^2^	109 (52.9%)	15 (41.7%)	94 (55.3%)	
Unknown	110	19	91	
Perineural invasion				NS
Yes	149 (60.8%)	29 (64.4%)	120 (60.0%)	
No	96 (39.2%)	16 (35.6%)	80 (40.0%)	
Unknown	71	10	61	
Regression				NS
Yes	8 (3.4%)	3 (7.0%)	5 (2.6%)	
No	229 (96.6%)	40 (93.0%)	189 (97.4%)	
Unknown	79	12	67	
LVI				NS
Yes	4 (1.7%)	1 (2.5%)	3 (1.6%)	
No	226 (98.3%)	39 (97.5%)	187 (98.4%)	
Unknown	86	15	71	

^a^Significant on univariable and multivariable logistic regression analysis for predicting nodal status

^b^Significant only on univariable logistic regression analysis for predicting nodal status

LVI: lymphovascular invasion; NS: not significant.

### Clinicopathologic characteristics correlated with nodal status

Node-positive patients were significantly younger compared with node-negative patients (median 62.6 vs. 69.4 years, p<0.05; Table 1). In addition, node-positive patients had significantly thicker tumors than node-negative patients (median 4.65 vs. 3.60 mm, p<0.01). Mixed histology was associated with a significantly higher proportion of node-positivity, with nodal metastases occurring in 24 of 90 patients with mixed DM (26.7%) versus 15 of 103 patients with pure DM (14.6%, p<0.05).

Univariable analysis showed that age, Breslow thickness and histologic subtype differed significantly between node-positive and node-negative patients (all p<0.05). Multivariable analysis demonstrated that younger age (OR: 0.97, 95% CI: 0.95–0.99; p<0.01) and mixed histology (OR: 2.33, 95% CI: 1.12–4.86; p<0.01) significantly predicted nodal metastasis. No significant differences in Clark level, margin status, ulceration, MR, PNI, regression or lymphovascular invasion were observed between node-positive and node-negative patients.

### Recurrence and recurrence-free survival

Overall, 295 patients (93%) were evaluable for recurrence, while 21 patients were lost to follow-up. Recurrent disease developed in 87 patients (29.5%), with 30 of these patients having an isolated local recurrence. Regional recurrence developed in 23 cases, with 22 patients having a nodal recurrence and one developing in-transit disease. Distant metastasis developed in 33 patients, and the site of recurrence was unknown for one patient.

**Fig 1:**
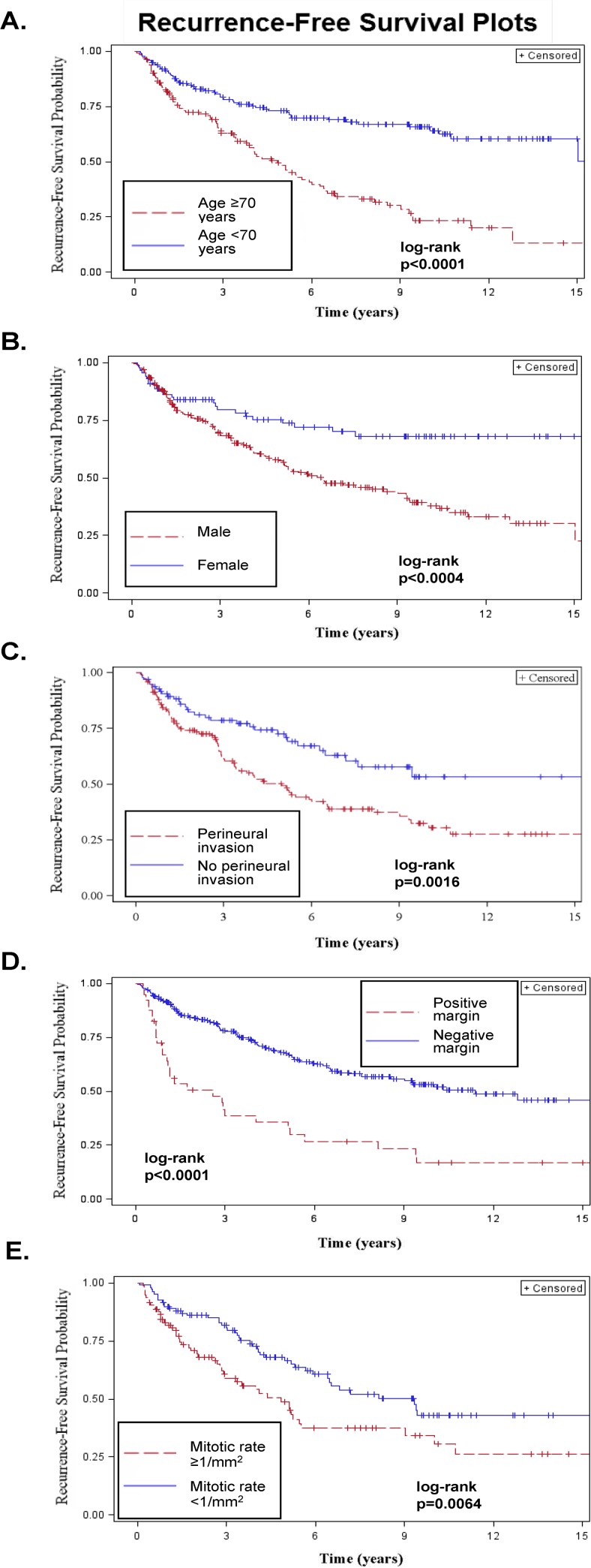
Impact of clinicopathologic factors on recurrence-free survival (RFS) in patients with clinically localized desmoplastic melanoma. A. RFS by age (≥70 vs. <70 years). B. RFS by gender. C. RFS by presence or absence of perineural invasion. D. RFS by final histologic margin status at the primary tumor site. E. RFS by mitotic rate (≥1/mm^2^ vs. <1/mm^2^).

Five-year RFS was 62.3% (95% CI: 56.6%–68.1%). Age, gender, Breslow thickness, Clark level, histologic subtype, ulceration, PNI, MR and margin status were associated with RFS on univariable analysis (all p<0.05). Multivariable analysis showed that older age (defined as ≥70 years), male gender, PNI, positive margin status and MR ≥1/mm^2^ significantly predicted worse RFS (all p<0.05, Table 2 and Fig. 1).

**Table 2:** Multivariable analysis for predictors of survival in patients with clinically localized desmoplastic melanoma.

	HR	95% CI	p-value
**Recurrence-free survival**			
Age ≥70 years	2.38	1.65–3.45	**<0.01**
Male gender	1.99	1.21–3.21	**<0.01**
Perineural invasion	1.78	1.14–2.78	**0.01**
Positive margin status	2.01	1.31–3.09	**<0.01**
Mitotic rate ≥1/mm^2^	1.66	1.09–2.53	**0.02**
**Overall survival**			
Age ≥70 years	2.84	1.89–4.26	**<0.01**
Male gender	2.61	1.47–4.62	**<0.01**
Breslow thickness >4 mm	1.49	1.00–2.20	**0.05**
Mitotic rate ≥1/mm^2^	1.61	1.01–2.57	**0.05**
**Melanoma-specific survival**			
Breslow thickness >4 mm	3.12	1.61–6.06	**<0.01**
Mitotic rate ≥1/mm^2^	3.39	1.43–8.05	**<0.01**
Positive nodal status	1.96	1.04–3.68	**0.04**

HR: hazard ratio, CI: confidence interval.

### Overall survival and melanoma-specific survival

Vital status was available in all 316 patients, with 111 (35.1%) known to have died. The cause of death was known in 79 cases, and was melanoma-related in 47 cases and not melanoma-related in 32 cases. Overall, 14.9% (47 of 316 patients) died from melanoma while 10.1% (32 of 316 patients) died from other causes and 10.1% (32 of 316 patients) died from unknown causes.

**Fig 2:**
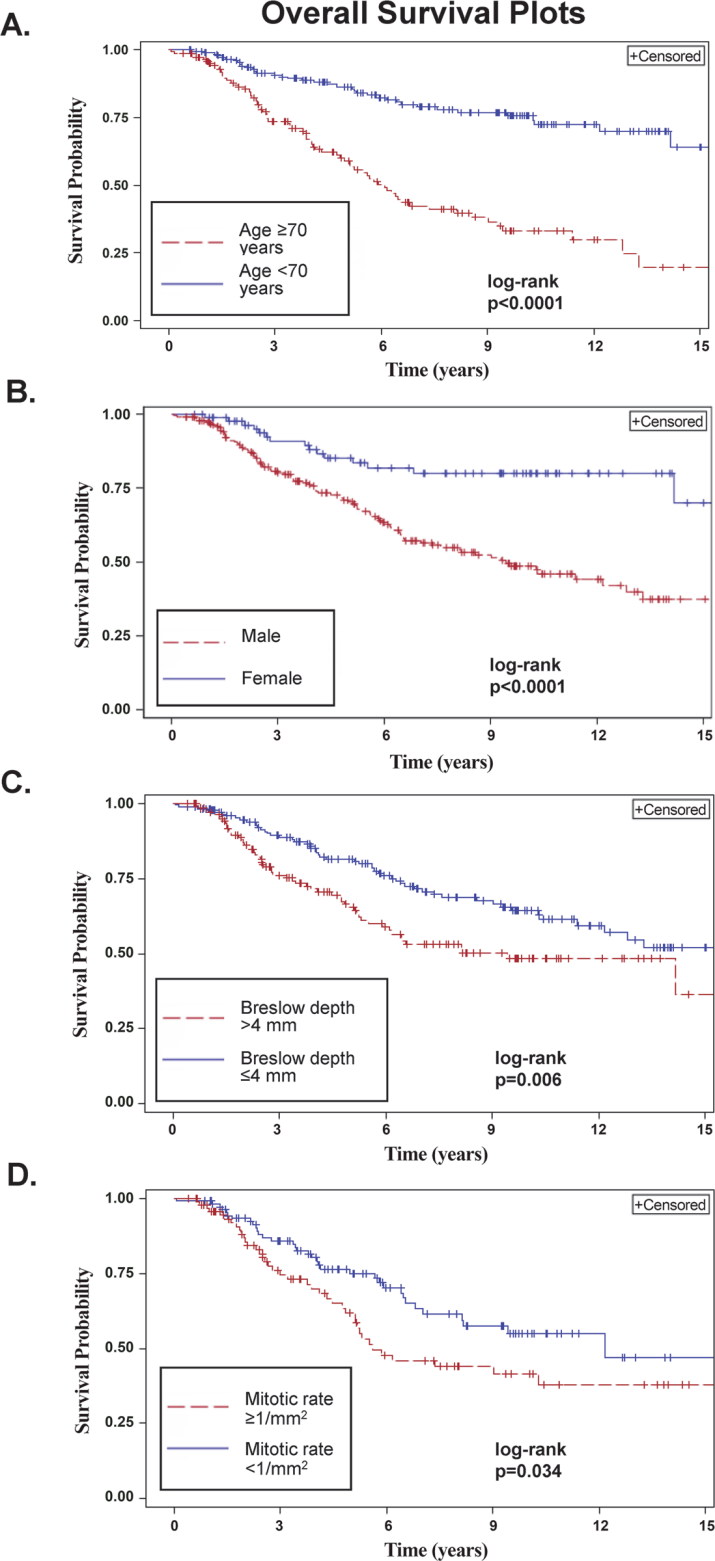
Impact of clinicopathologic factors on overall survival (OS) in patients with clinically localized desmoplastic melanoma. A. OS by age (≥70 vs. <70 years). B. OS by gender. C. OS by Breslow thickness (≤4 mm vs. >4 mm). D. OS by mitotic rate (≥1/mm^2^ vs. <1/mm^2^).

Five-year OS was 74.5% (95% CI: 69.2%–79.7%). Age, gender, Breslow thickness, Clark level, histologic subtype, ulceration, PNI, MR and margin status correlated with OS on univariable analysis (all p<0.05). Multivariable analysis showed that older age, male gender, Breslow thickness >4 mm and MR ≥1/mm^2^ significantly predicted worse OS (all p≤0.05, Table 2 and Fig. 2).

**Fig 3:**
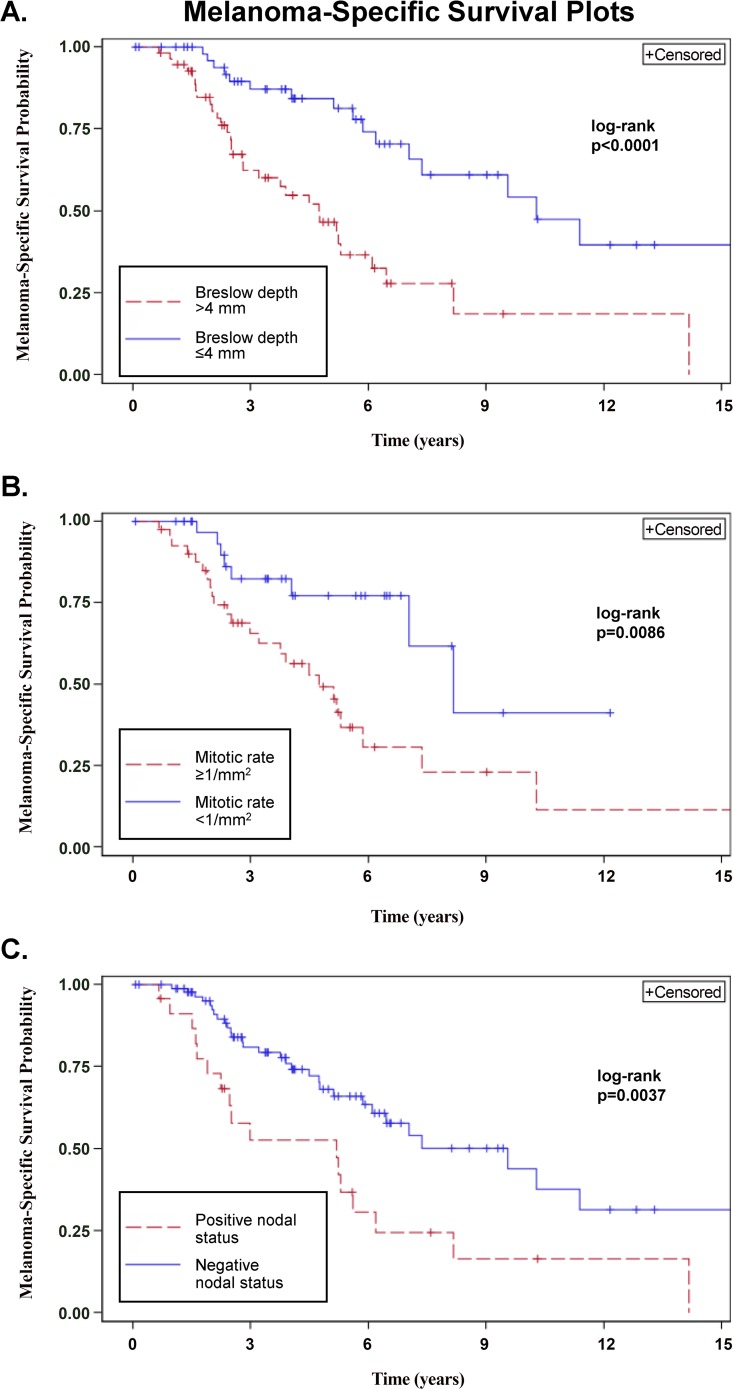
Impact of clinicopathologic factors on melanoma-specific survival (MSS) in patients with clinically localized desmoplastic melanoma. A. MSS by Breslow thickness (≤4 mm vs. >4 mm). B. MSS by mitotic rate (≥1/mm^2^ vs. <1/mm^2^). C. MSS by nodal status.

Five-year MSS was 65.1% (95% CI: 55.0%–75.3%). Age, Breslow thickness, Clark level, MR and nodal status were correlated with MSS on univariable analysis (all p<0.05). Multivariable analysis demonstrated that Breslow thickness >4 mm, MR ≥1/mm^2^ and a positive nodal status significantly predicted worse MSS (all p<0.05, Table 2 and Fig. 3).

## Discussion

Desmoplastic melanoma is a rare variant of cutaneous melanoma. In particular, DM has been associated with substantial uncertainty regarding prognosis as well as the risk of regional nodal metastasis. This uncertainty has resulted in confusion and conflict in terms of recommendations for staging and treatment. The reported rate of nodal metastasis for DM in contemporary series ranges from 0–18.8%, with a higher rate reported for mixed tumors [7–20]. Our study found a nodal metastasis rate of 17.4%; this rate was significantly higher for mixed compared with pure DM (26.7% vs. 14.6%, p<0.01 on multivariable analysis). However, the rate of nodal metastasis in pure DM, while lower than in mixed tumors, was still substantial in our experience. Furthermore, our results are also consistent with other melanoma studies that have correlated younger age with a higher nodal metastasis rate [22, 23].

There is also uncertainty in the literature about the prognostic significance of nodal metastasis in DM. The primary purpose of the current study was to evaluate clinicopathologic factors prognostic for survival in patients with DM. Since there is suggestion in the literature that DM may behave clinically in a manner that is different from non-DM (e.g. lower propensity for nodal metastasis), we wanted to determine if the same prognostic factors for survival in patients with non-DM were also seen in patients with DM. Nodal status in DM patients was reported to predict MSS in a SEER-based study and in a case-matched study,[4, 12] and to predict OS in a retrospective analysis [9]. We found that nodal status was a significant predictor of MSS but not of OS on multivariable analysis. The lack of impact of nodal status on OS is undoubtedly partly attributable to the high number of non-melanoma related deaths that occurred in this relatively older population (median age 68 years). Interestingly, although histologic subtype predicted nodal status, it did not predict MSS. This would imply that, although nodal metastasis is more frequently seen in mixed tumors, once lymph node spread occurs it follows a similar course regardless of histologic subtype. Collectively, these findings have important implications for the management of DM, where the use of SLNB for staging has been controversial, especially for tumors of pure histologic subtype. Our prior study evaluated predictive factors for a positive SLN and the prognostic significance of SLN status in patients with DM [7]. We found that SLN status predicted melanoma-specific mortality and that histologic subtype significantly predicted SLN metastasis after adjusting for age but the rate of SLN disease was high enough in both mixed and pure variants to warrant SLNB for both subtypes [7]. The data from the current study would also suggest that SLNB should be used for DM patients with either histologic subtype, unless patient age or comorbidities would limit the value of the prognostic information obtained from characterizing a patient’s nodal status.

In addition to nodal status, our results demonstrated that Breslow thickness >4 mm and MR ≥1/mm^2^ predicted significantly worse MSS and OS on multivariable analysis, while older age and male gender were associated with worse OS and RFS but not worse MSS. Several studies have evaluated additional factors influencing survival in DM patients. Breslow thickness has been shown to predict survival in DM patients [4, 5, 12, 15]. Older age has also been correlated with worse OS in one study and with worse MSS in a SEER-based study [9, 14]. DM patients represent an older population, and in our study, a considerable proportion of deaths with known causes were not due to melanoma, so it is not surprising that older age would be correlated with worse OS. Murali et al. also reported that gender significantly predicted OS, [24] and two studies have correlated MR with survival [5, 16]. In contrast to prior studies that have reported a significant association between histologic subtype and RFS or MSS, [2, 3, 8] our study did not find histologic subtype to be a significant predictor of RFS, MSS or OS on multivariable analysis.

Margin status and Clark level have also been reported to predict RFS in DM [2, 9]. In our study, margin status but not Clark level was significantly associated with RFS on multivariable analysis. A study on head and neck DM showed that PNI correlated with worse RFS on univariable analysis [16]. Similarly, we found PNI to be a significant predictor of RFS on both univariable and multivariable analysis. Interestingly, MR ≥1/mm^2^ was also a significant independent predictor of RFS in our study.

## Conclusions

In summary, younger age and a mixed histologic subtype significantly predict nodal metastases in DM patients, however nodal disease is still seen in a considerable proportion of pure DM patients (14.6%). For DM patients, nodal status significantly predicts MSS, although it does not predict OS. Other factors that significantly predict MSS in DM patients include MR and Breslow thickness. Thus, we believe that sentinel lymph node evaluation potentially has an important role in the management of all DM patients [7]. Finally, DM patients represent an older population, and a considerable proportion of deaths with known causes are not due to melanoma, so comorbidities should be carefully considered in making staging and treatment decisions.
